# Genome-Wide Identification of NBS-LRR Family in Three *Nicotiana* Genomes and Their Expression During Disease Resistance

**DOI:** 10.3390/genes16060680

**Published:** 2025-05-30

**Authors:** Ying Liu, Wenjing Liu, Haozhe Huang, Caixia Zhang, Long Yang

**Affiliations:** 1 College of Plant Protection, Shandong Agricultural University, Taian 271018, China; 15839772009@163.com (Y.L.); lwj030208@163.com (W.L.); 2 College of Agronomy, Shandong Agricultural University, Taian 271018, China

**Keywords:** NBS-LRR gene family, *Nicotiana tabacum*, phylogenetic analysis, disease resistance

## Abstract

**Background:** The NBS-LRR gene family plays a critical role in plant disease resistance and is considered a key determinant of plant immune responses. Research on the NBS-LRR gene family has grown rapidly, with significant progress driven by advances of molecular biology techniques. However, to date, there has been no systematic identification of NBS-LRR genes in *Nicotiana* species. **Methods**: In this study, we systematically characterized the NBS gene families in three *Nicotiana* genomes, investigated the evolution and environmental selection during the species formation, and explored the key NBS genes involved in disease resistance. **Results**: Results showed that 1226 NBS genes are present across the three *Nicotiana* genomes, and 76.62% of the members in *Nicotiana tabacum* could be traced back to their parental genomes. In addition, whole-genome duplication was found to contribute significantly to the expansion of NBS gene families. In addition, many NBS genes associated with disease resistance were identified, including one multi-disease resistance gene. **Conclusions**: This study provides new insights into the formation of NBS gene families in *Nicotiana* and offers new clues for understanding plant immunity.

## 1. Introduction

Disease resistance in plants is a complex process, and resistance genes serve as the foundation for the formation of plant resistance and are also the specific determinants of plant immune responses [1]. To date, more than 300 disease resistance genes from different plants have been identified, of which 60% belong to the nucleotide-binding site–leucine-rich repeat (NBS-LRR) gene family [2,3]. Therefore, the NBS family is an important class of disease resistance genes in plants and has been extensively studied across diverse plant species. The proteins encoded by NBS genes usually contain the nucleotide-binding site domain and leucine-rich repeat sequence (LRR), which can trigger plant immune response by recognizing pathogen-effector proteins and play a vital role in enhancing adaptability to both biotic and abiotic stresses [4].

The NBS-LRR gene family can be divided into different subfamilies based on the conserved domains of NBS-LRR genes in several ways. According to the domains contained in the N-terminal and C-terminal, the NBS-LRR gene family can be divided into eight subfamilies, including CC-NBS (CN), CC-NBS-LRR (CNL), NBS (N), NBS-LRR (NL), RPW8-NBS (RN), RPW8-NBS-LRR (RNL), TIR-NBS (TN), and TIR-NBS-LRR (TNL) [5,6]. In addition, there are still several other different ways to classify it, such as the NBS-LRR gene family of *Solanaceae*, which is split into TNL (TIR-NBS-LRR) and non-TNL (non-TIR-NBS-LRR) sub-families depending on the presence or absence of the TIR domain in the N-terminal [7]. Additionally, based on the N-terminal domain, the *Brassicaceae* NBS-LRR gene family was divided into TIR-NBS-LRR (TNL), CC-NBS-LRR (CNL), and RPW8-NBS-LRR (RNL) subfamilies [8].

Research on the NBS-LRR gene family has expanded rapidly and great progress has been made with significant progress driven by advancements in molecular biology. Studies have revealed extensive variation in NBS-LRR gene count across plant species, with 73 NBS-LRR genes found in *Akebia trifoliata* [9], 167 in *Dioscorea rotundata* [10], 352 in *Vitis vinifera* [11] and 2151 in *Triticum aestivum* [12]. Vast numbers of NBS-LRR genes have been identified and confirmed functionally in varied plant species, which is mainly due to the completion of genome sequencing of these species. Significant progress also has been made in the functional mechanism of NBS-LRR family members; for example, studies on the sweet orange found that the NBS-LRR genes are generated via gene duplication events, with distinct conserved domains exhibiting specialized functions within NBS-LRR genes [1]. The nucleotide-binding site (NBS) domain primarily mediates signal transduction [13], while the leucine-rich repeat (LRR) domain often plays an important role in protein interactions and is responsible for specific recognition [14]. Li et al. confirmed that silencing the NBS-LRR gene reduced resistance to *Verticillium dahlia* in cotton [15]. Xu et al. clarified that heterologous expression of the maize NBS-LRR gene could improve resistance to *Pseudomonas syringae pv.* in *Arabidopsis thaliana* [16]. Xun et al. reported that overexpression of the soybean TNL gene conferred broad-spectrum resistance to viral pathogens in soybean [17].

*Nicotiana tabacum*, because of its easy cultivation, rapid growth, and amenability to genetic transformation and gene editing, has become an ideal model plant for disease resistance studies in plants. As a model plant for disease resistance research, tobacco has irreplaceable value in plant disease resistance mechanism analysis and breeding owing to its genomic characteristics, abundant disease resistance gene resources, and efficient research tools. Recent studies have shown that efficient genome editing tools suitable for dicotyledonous plants with new promoters could significantly improve gene editing efficiency; their homozygous mutation rate is close to 100%, and regeneration cycle is shortened [18]. This technological breakthrough accelerates the research and application of the function of tobacco disease resistance genes.

*N. tabacum* is an allotetraploid, formed via diploidization following hybridization of *N. sylvestris* and *N. tomentosiformis*. Hence, the research of NBS family genes will not only deepen the understanding of the plant immune system and provide scientific basis for breeding resistant varieties but also provide the evolutionary history of the NBS genes family or even the genome of *N. tabacum.*

In this study, our objectives were to characterize the NBS genes families across three *Nicotiana* genomes and measure the evolution and environment selection during the species divergence. In addition, we also sought to explore key NBS genes which were involved in disease resistance. This study will provide new insights into the formation of NBS families in *Nicotiana* and supply new clues for plant immunity research.

## 2. Materials and Methods

### 2.1. Identification and Classification of NBS Genes in Three Nicotiana Genomes

The genome assemblies and annotated protein sequences of *N. tabacum*, *N. sylvestris*, and *N. tomentosiformis* were downloaded from Zenodo, with accession numbers 8256256, 8256252, and 8256254 [19]. For comprehensive identification of NBS-LRR family members, we performed hidden Markov model (HMM) searches using HMMER v3.1b2 [20] with the model PF00931 from the PFAM database. The TIR and LRR domains were identified using PFAM domains (PF01582, PF00560, PF07723, PF07725, PF12779, PF13306, PF13516, PF13855, PF14580, PF03382, PF01030, PF05725). The Coiled coil (CC) domains were confirmed via the NCBI Conserved Domain Database (CDD) (https://www.ncbi.nlm.nih.gov/cdd, accessed on 18 March 2025) [21], and the completeness of all domains was also confirmed by the NCBI conserved domain function.

### 2.2. Phylogenetic and Duplication Analysis of NBS Families

Multiple sequence alignment of NBS-LRR protein sequences was performed using MUSCLE v3.8.31 [22] with default parameters. MEGA11 were used to construct the NG tree with a bootstrap of 1000. The whole-genome duplication in three *Nicotiana* genomes was first analyzed using self-BLASTP [23], based on their own protein sequences. Then, the segment duplication and tandem duplication across the whole genome were processed by MCScanX [24] under default configurations. Syntenic blocks across genomes were determined through reciprocal BLASTP searches (-s 100 parameter for scoring matrix optimization) followed by MCScanX-based collinearity detection. Syntenic genes were obtained from the collinearity file of MCScanX; the paired gene were processed with ParaAT [25]; and selection pressures were quantified by calculating non-synonymous (Ka) and synonymous (Ks) substitution rates with KaKs_Calculator 2.0 [26] with the evolutionary model of Nei-Gojobori (NG).

### 2.3. RNA-Seq Analysis

The RNA-seq datasets for two disease resistances (black shank and bacterial wilt) in *N. tabacum* were downloaded from the NCBI SRA, accession numbers SRP310543 [27] and SRP141439. Raw sequencing files (in SRA format) were converted to the FASTQ format using fastq-dump v2.6.3 [28]. Read quality control was performed using Trimmomatic v0.36 [29] with the minimum reads length of 90 bp. The cleaned data were mapped onto the reference genome of *N. tabacum* by Hisat2 [30]. Transcript quantification and differential expression analysis were conducted using Cufflinks v2.2.1 [31] with FPKM normalization. Differentially expressed genes (DEGs) were identified through Cuffdiff [31].

## 3. Results

### 3.1. Identification of the NBS Family in the Genomes of Three Nicotiana Species

In this study, we identified the NB-ARC domain of three *Nicotiana* species (*N. tabacum*, *N. sylvestris*, and *N. tomentosiformis*) based on the hidden Markov model (HMM), with the model of PF00931. And all these members were then scanned against the NCBI Conserved Domain Database (CDD); only genes containing the associated domains were retained. In total, we identified 1226 NBS genes in the tree genomes; there were 344 NBS members in *N. sylvestris*, a little more than *N. tomentosiformis* (279). The *N. tabacum* contained the most NBS members (603), accounting for approximately the combined total of its parental gene count (Table 1 and Table S1).

All the identified members were also classified by their domain composition. Approximately 45.5% of genes in *Nicotiana* contained only the NBS domain, followed by CC-NBS (23.3%), while TIR-NBS members were the least abundant, accounting for only 2.5% of the entire family (Table 1 and Table S1).

### 3.2. Phylogenetic Analysis of NBS Families

**Figure 1:**
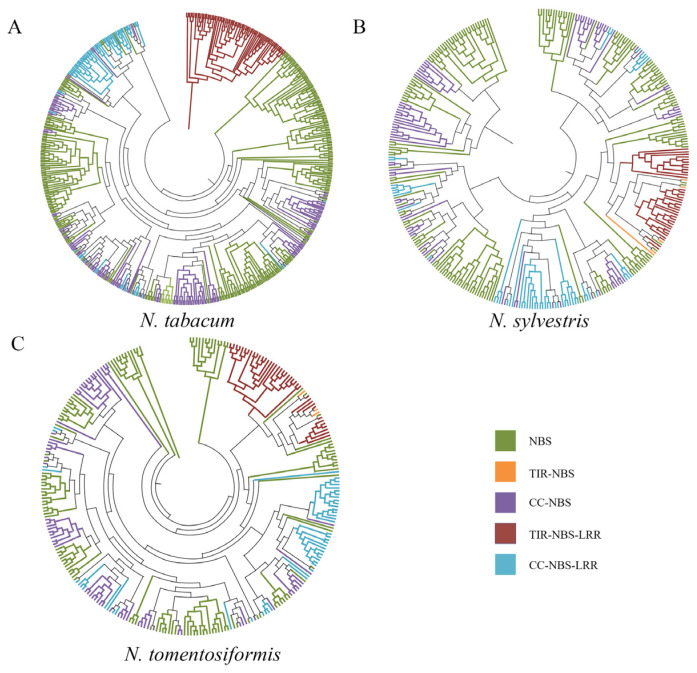
Phylogenetic analysis of NBS families of three *Nicotiana*. (**A**) Phylogenetic analysis of NBS families of *N. tabacum*; (**B**) phylogenetic analysis of NBS families of *N. Sylvestri*; (**C**) phylogenetic analysis of NBS families of *N. tomentosiformis*.

Based on the protein sequences, we constructed the phylogenetic trees of this genes family in three *Nicotiana*, a neighbor-joining (NJ) tree of all members was generated by MEGA with 1000 bootstrap replicates. The result indicated that the sub-families were relatively conserved, with most genes belonging to one sub-family clustering into shared branches, especially the TIR-NBS family which exhibited high conservation. In addition to members containing CC or TIR domains, the members containing only NBS domains were the largest sub-family and also could be divided into several groups (Figure 1).

As it is usually believed that *N. tomentosiformis* and *N. sylvestris* are two parents of *N. tabacum*, we investigated the member relationships between the parents and *N. tabacum.* Results showed that 76.62% of *N. tabacum* family members were inherited from their progenitors, with gene loss and gain occurring during the *N. tabacum* formation. Specifically, about 82 genes were lost from the *N. sylvestris* sub-genome, and 65 genes were lost from the *N. tomentosiformis* sub-genome, while *N. tabacum* acquired 141 NBS genes through duplications, transposable element (TE)-mediated events, or other mechanisms.

### 3.3. Duplication and Selection of NBS Families in Nicotiana

**Figure 2:**
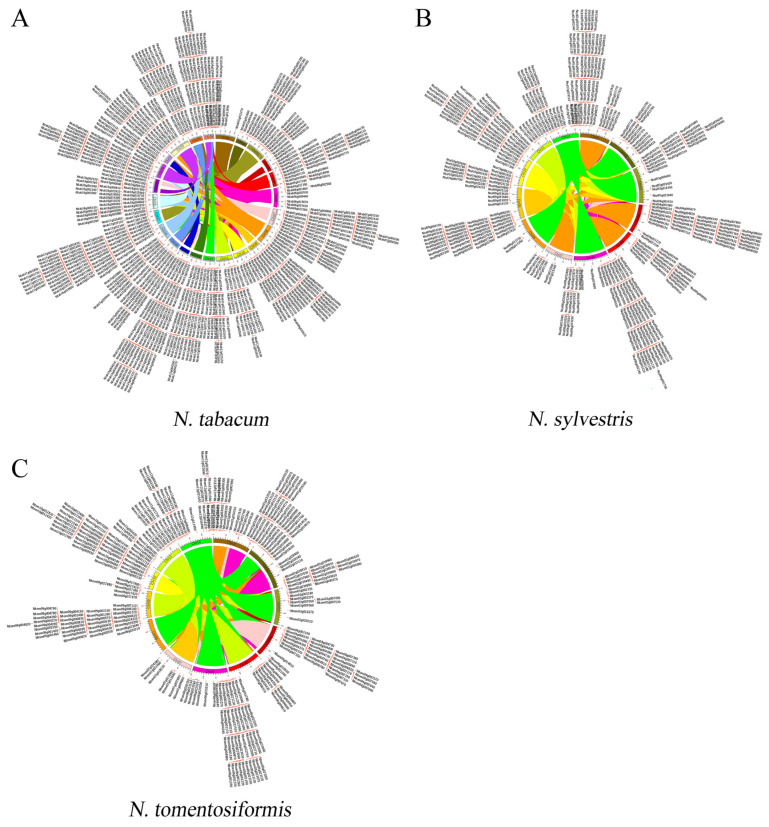
Segment duplication events involved the NBS family in the tree *Nicotiana*. (**A**) Segment duplication events occurred in *N. tabacum*. (**B**) Segment duplication events occurred in *N. sylvestris*. (**C**) Segment duplication events occurred in *N. tomentosiformis.* The genes id outside the circle is the distribution of NBS-LRR genes in this genome, The links of different chromosomes were the segment duplication events during the evolution.

Ks distribution analysis indicated that NBS genes family in tobacco experienced a complex series of duplication, including the WGT event which occurred ~120–150 Mya. Besides this duplication, at least one duplication event contributed to diploidization of *N. tomentosiformis* and *N. sylvestris*. In total, the duplication of NBS families were involved in a huge number of segment duplication events; in *N. tomentosiformis*, NBS families were involved in 16,335 segment duplication and 147 tandem duplication events (Figure 2A). In *N. sylvestris*, the NBS family was involved in 18,548 segment duplication and 190 tandem duplication events (Figure 2B). However, in *N. tabacum*, the number of NBS family members was nearly the sum of its parents, suggesting that terminal duplication or hybridization events drove NBS family expansion in *N. tabacum* (Figure 2C). These findings indicated that segment and tandem duplication contributed greatly to the formation of NBS families in *Nicotiana*.

**Figure 3:**
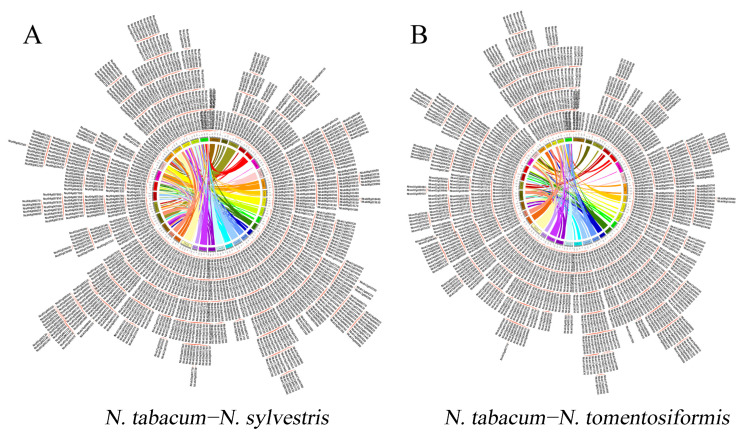
Synteny analysis of NBS family members between *N. tabacum* and its two parental species. (**A**) Synteny family analysis members between *N. tabacum* and *N. sylvestris*. (**B**) Synteny family analysis members between *N. tabacum* and *N. tomentosiformis*. The gene IDs outside the circle represent the distribution of NBS-LRR genes across the genome. The links between different chromosomes indicate the segment synteny between the parents and *N. tabacum*.

**Figure 4:**
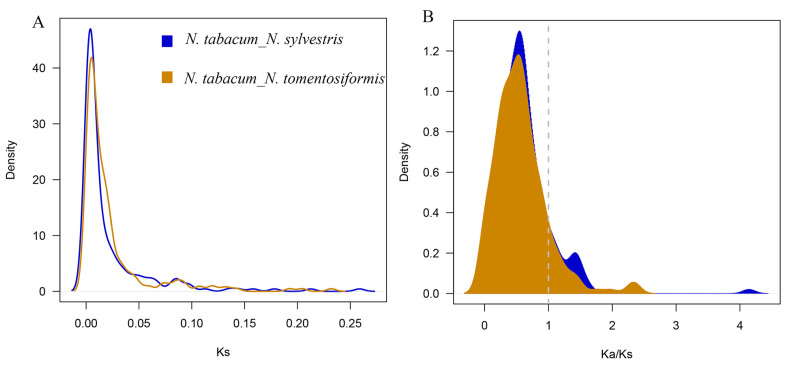
Ks and Ka/Ks analysis of NBS pairs between the *N. tabacum* and its parents. (**A**) Ks value distribution of NBS members. (**B**) Distribution of Ka/Ks value between *N. tabacum* and its parents.

The parent–offspring NBS genes between the two parents and *N. tabacum* were re-analyzed to finalize the formation of NBS families in *N. tabacum*. Based on blast and syntenic analyses, 11,009 pairs between *N. tomentosiformis* and *N. tabacum*, 15,489 pairs between *N. sylvestris* and *N. tabacum* were identified (Figure 3). The Ks distribution analysis indicated that these members of *N. tabacum* and *N. sylvestris* divergence at the Ks value of 0.0036 (~204.21 kya) and *N. tabacum* and *N. sylvestris* divergence at 0.0045 (~257.59 Kya) (Figure 4A). To explore the impact of the environmental selection on the evolution of this family, the Ka/Ks values were also considered; in *N. tomentosiformis*, Ka/Ks values of 176 (88.89%) gene pairs were lower than 1, with the average value of 0.4617. Only 11.11% of gene pairs contained the Ka/Ks value higher than 1. In *N. sylvestris*, Ka/Ks ratios of 168 (86.15%) gene pairs were lower than 1, the average value being about 0.4728. Only 13.85% gene pairs contained the Ka/Ks value higher than 1 (Figure 4B). These results imply that after the formation of *N. tabacum*, the genes of NBS families undergo purifying selection, meaning that their functions remained stable and similar to those of their orthologs in the parental species.

### 3.4. Expression and Behavior of NBSs Under Black Shank and Bacterial Wilt

**Figure 5:**
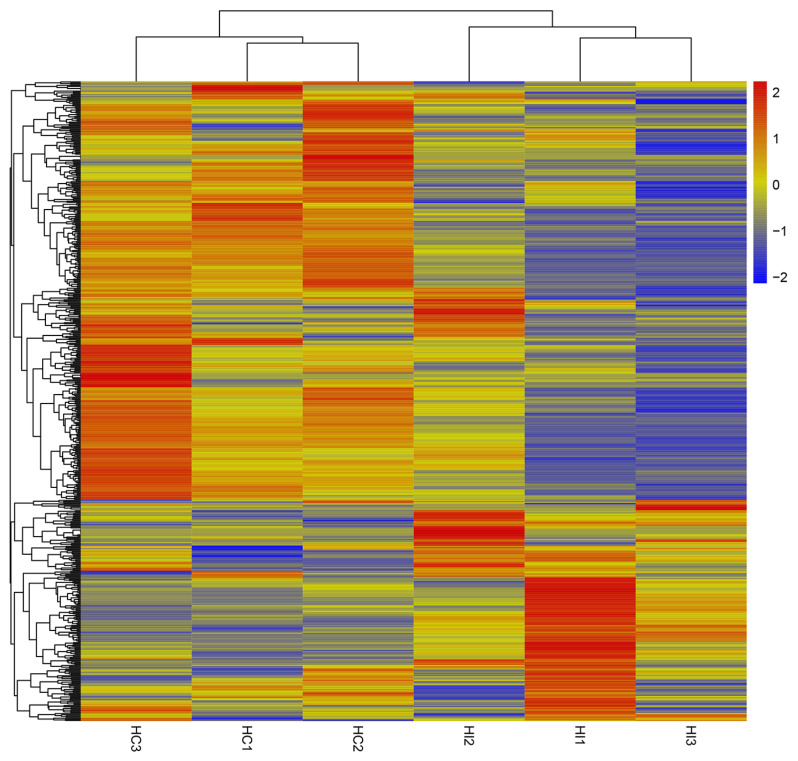
Expression pattern of DEGs under black shank diseases. The expression level was normalized by Z-score methods. HC is the blank control, HI is the pathogen-infected treatment, each sample have three biological replicates.

In this study, the expression and behavior of NBS genes under black shank and bacterial wilt were analyzed in tobacco. The online transcriptome (SRP310543) was used to identify the differently expressed genes (DEGs) under black shank; 13,483 DEGs were identified, including 99 NBS genes, of which39 were up-regulated (Table S2) and 60 were down-regulated. Notably, the most significant upregulation gene was Ntab14g000450, and the Log2(Fold Change) was 3.20552, while the most significant downregulation gene was Ntab00g003660, and the Log2(Fold Change) was −3.92828 (Figure 5). Additionally, homology analysis showed that 46 of 99 NBS genes were from *N. tomentosiformis* and 45 were from *N. sylvestris*.

**Figure 6:**
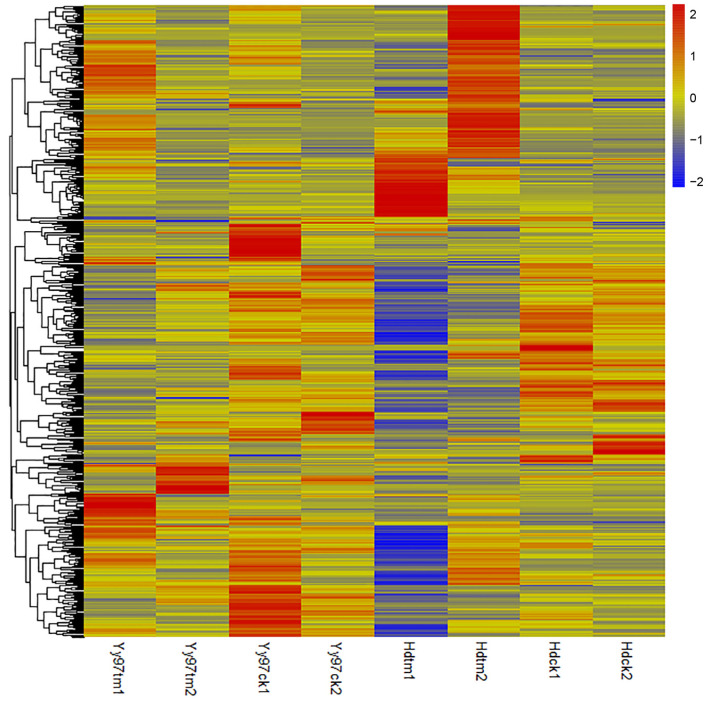
Expression pattern of DEGs under bacterial wilt diseases of Hd and Yy97. The expression level was normalized by Z-score methods. Ck is the blank control; tm is the pathogen-infected treatment; each sample has with two biological replicates.

As for the bacterial wilt, transcriptome resistance analysis (SRP141439) was performed in two tobacco varieties, and 20,194 DEGs were identified in Hd (*N. tabacum* cultivar: Honghua Dajinyuan). In total, 189 NBS genes were found in the DEGs; 135 were up-regulated (Table S3), and 54 were down-regulated. The most significantly upregulated genes wereNtab03g028090, Ntab05g002750, and Ntab08g007540. The expression of these three genes changed from non-expression to expression. In contrast, the most significantly downregulated gene was Ntab07g000910, with a Log2(Fold Change) of −5.05785 (Figure 6). Homology analysis showed that 85 of the 189 NBS genes originated from *N. tomentosiformis* and 84 from *N. sylvestris*.

In Yy97 (*N. tabacum* cultivar: Yunyan97), 9465 DEGs were identified, including 81 NBS genes. Among these genes, 54 NBS genes were up-regulated (Table S4) and 27 down-regulated. The most significantly up-regulated gene was Ntab03g001370, and the Log2(Fold Change) was 6.69872. Meanwhile, the most significantly down-regulated gene was Ntab07g016420, with a Log2(Fold Change) of −2.36381 (Figure 6). Homology analysis showed that 34 of the 81 NBS genes were from *N. tomentosiformis* and 38 were from *N. sylvestris.*

**Figure 7:**
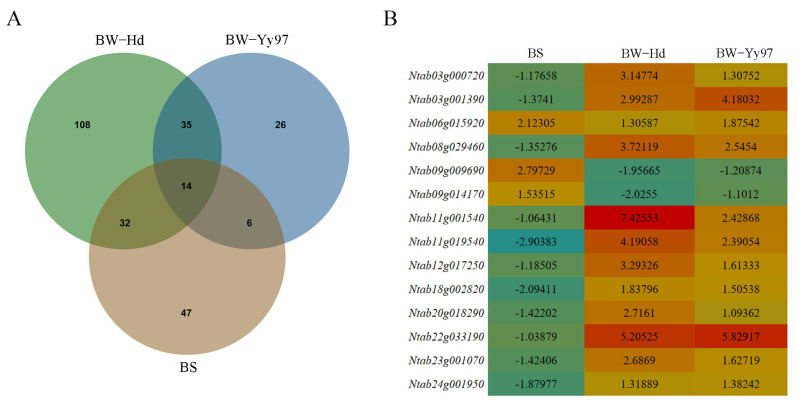
Identification of 14 shared genes and their expression pattern during disease infection. (**A**) Venn diagram of differential expression genes of black shank and bacterial wilt in Hd and Yy97. (**B**) Log2(Fold Change) of 14 shared genes under black shank and bacterial wilt.

Our results showed that 14 NBS DEGs were shared by the two varieties of black shank and bacterial wilt, and these genes accounted for 2.32% of all NBS families (603) (Figure 7A). Homology analysis showed that these 7 of 14 NBS genes were from *N. tomentosiformis* (Ntab03g001390, Ntab06g015920, Ntab11g019540, Ntab08g029460, Ntab22g033190, Ntab20g018290, Ntab03g000720), and the other 7 NBS genes were from *N. sylvestris* (Ntab18g002820, Ntab23g001070, Ntab12g017250, Ntab11g001540, Ntab24g001950, Ntab09g009690, Ntab09g014170). Only one gene (Ntab06g015920) of the 14 NBS DEGs was up-regulated according to the comprehensive analysis of two kinds of pathogens resistance genes, while two upregulated genes (Ntab09g009690, Ntab09g014170) were found only in black shank disease, and eleven upregulated genes were found only in bacterial wilt. Among these genes, the most significantly upregulated gene was Ntab11g001540 with the Log2(Fold Change) of 7.426 (Figure 7B).

## 4. Discussion

### 4.1. The Disease Resistance of N. tabacum Mainly Inherited from Its Wild Parents

NBS genes played an important role in the disease resistance of plants and is central to the plant immune system. In this study, we identified the NBS family members in *N. tabacum* and its two parents (Table 1); the phylogenetic tree and syntenic analysis indicated that most NBS genes in *N. tabacum* were obtained from the parents (Figure 1). In addition, the Ks distribution of these orthologous genes indicated this combination of two parents occurred about 200–250 Kya (Figure 4A), which is in accordance with previous studies [32]. After this divergence, the Ka/Ks values of most orthologous genes were found to be lower than 1 (Figure 4B), which implied that most orthologous genes have been subject to purifying selection and have remained conserved throughout evolutionary history. The lower Ka values further imply functional importance, as amino acid changes in these genes may lead to maladaptive consequences in response to environmental pressures or disease. Therefore, the gene function of NBS in *N. tabacum* was similar to that of their parents. However, synteny and gene count analyses across the three species indicate that both gene loss and gene gain also occurred, although there was no evidence that this loss or gain genes take part in disease resistance, these contribute to the diversification of NBS genes family in *N. tabacum*. Meanwhile, considering the function of NBS-LRRs and the loss of resistance during the formation, hybridization with wild species is a meaningful choice for resistance improvement.

### 4.2. NBS Genes May Play an Important Role in Disease Resistance

In this study, we identified a huge number of DEGs associated with responses to black shank and bacterial wilt. Specifically, 99 NBS genes were responsive to black shank, and 189 (Hd) and 81 (Yy97) NBS genes responded to bacterial wilt. Although numerous NBS genes responded to disease infection, only one-third of the DEGs were up-regulated in response to the black shank infection, while most of these genes were down-regulated. This pattern suggests that many NBS-LRR genes may not participate directly in resistance to black shank, but instead could be involved in responses to other pathogens or biological processes. The down-regulation of these genes implied that most NBS genes are pathogenic or functionally specific, where not all genes took contribute to resistance against a particular disease. During the bacterial wilt infection, we found great differences between two cultivars. Yy97 has fewer DEGs than Hd; only about half NBS DEGs were identified in Yy97. Therefore, distinct NBS genes family members respond to different pathogens and that gene expression dynamics can vary significantly between different cultivars or plants. When compared to two diseases, we obtained one NBS gene (Ntab06g015920) that was up-regulated during all diseases, and the detailed annotation of this gene indicated that this is also an ABS transporter type C which was related to disease resistance. All this suggests that this NBS gene has multi-disease resistance. These genes provide an overview of the behavior of the NBS-LRR family under infection. Further validation and marker development of these candidate genes could significantly advance disease resistance breeding efforts.

## 5. Conclusions

In this study, we identified 603 (*N. tabacum*), 344 (*N. sylvestris*), 279 (*N. tomentosiformis*) NBS genes in three *Nicotiana* species. The analysis indicates that whole genome duplication is a primary contributor to the expansion of NBS gene families is whole-genome duplication. Comparative Ka/Ks analysis between the *N. tabacum* and its parental species suggests that their functions had remained stable during the long evolution history. Finally, by transcriptome analysis, one NBS gene (Ntab06g015920) demonstrating multi-disease resistance was identified. Our study could offer novel insights into the NBS gene families in *Nicotiana* and contribute new insights into the genetic basis of plant immunity.

## Figures and Tables

**Table 1:** Detailed information of NBS families in three *Nicotiana* species.

	*N. tomentosiformis*	*N. sylvestris*	*N. tabacum*
NBS	127	172	306
TIR-NBS	7	5	9
CC-NBS	65	82	150
TIR-NBS-LRR	33	37	64
CC-NBS-LRR	47	48	74
Total	279	344	603

## Data Availability

Data are contained within the article or Supplementary Materials.

## Supplementary Materials

The following supporting information can be downloaded at: https://www.mdpi.com/article/10.3390/genes16060680/s1, Table S1: Genes and domains of NBS families in three *Nicotiana* species; Table S2: Function annotation of up-regulated NBS-LRR genes under black shank; Table S3: Function annotation of up-regulated NBS-LRR genes of Hd under bacterial wilt; Table S4: Function annotation of up-regulated NBS-LRR genes of Yy97 under bacterial wilt.
